# Electrostatic Precipitation of Submicron Particles in a Molten Carrier

**DOI:** 10.3390/pharmaceutics11060276

**Published:** 2019-06-13

**Authors:** Adrian Dobrowolski, Damian Pieloth, Helmut Wiggers, Markus Thommes

**Affiliations:** Laboratory of Solids Process Engineering, Department of Biochemical and Chemical Engineering, TU Dortmund University, 44227 Dortmund, Germany; adrian.dobrowolski@tu-dortmund.de (A.D.); damian.pieloth@tu-dortmund.de (D.P.); helmut.wiggers@tu-dortmund.de (H.W.)

**Keywords:** electrostatic precipitation, spray drying, submicron particles, solubility, bioavailability, nanoparticles, solid dispersion

## Abstract

Recently, submicron particles have been discussed as a means to increase the bioavailability of poorly water-soluble drugs. Separation of these small particles is done with both fibre and membrane filters, as well as electrostatic precipitators. A major disadvantage of an electrostatic precipitator (ESP) is the agglomerate formation on the precipitation electrode. These agglomerates frequently show low bioavailability, due to the decreased specific surface area and poor wettability. In this work, a new melt electrostatic precipitator was developed and tested to convert submicron particles into a solid dispersion in order to increase the bioavailability of active pharmaceutical ingredients. The submicron particles were generated by spray drying and transferred to the ESP, where the collection electrode is covered with a melt, which served as matrix after solidification. The newly developed melt electrostatic precipitator was able to collect isolated naproxen particles in a molten carrier. A solid naproxen xylitol dispersion was prepared, which showed a reduction of the dissolution time by 82%, and a release of 80% of the total drug, compared to the physical mixture.

## 1. Introduction

Poorly water-soluble active pharmaceutical ingredients are currently creating a challenge for bioavailability. Approximately 90% of the active ingredient molecules under development have poor water solubility [1]. The micronization of active ingredient (API) particles by milling or spray drying can be correlated with an increase in bioavailability [2]. The enlargement of the particle surface can lead to an increased mass transfer rate [3]. At the same time, the saturation concentration can be increased by the use of submicron particles [4].

Submicron particles are commercially produced in small scale laboratory equipment such as the Büchi B-90 HP. [5] Here, electrostatic precipitators (ESP) are used to collect spray-dried particles due to their high separation efficiency and the convenient particle recovery. The dispersed particles are charged by a corona discharge and deflected by an electric field towards the collecting electrode, where they are deposited upon impact. The separated particles form a particle layer of submicron particle agglomerates. [6] With regard to the processability of submicron particle powders, these agglomerates counteract the increase in bioavailability by reducing the size of the particles. For example, an agglomerate of many small particles is much more difficult to wet than a single particle [7].

Even in agglomerated form, nanopowders pose a hazard to the environment. Dispersed nanopowders create a higher risk of ignition, explosion, and pulmonary mobility. Electrostatic precipitators are more suitable for submicron particles than fiber filters because the valuable product is not trapped in the depths of the filter material. In the context of this work a new melt electrostatic precipitator (MESP) was developed following the nanosafe-by-design approach to trap the potentially dangerous substance directly in a carrier during the production process [8]. The creation of safe carriers containing potentially harmful drugs reduces redispersion of submicron particles in the air, and inhalation during product handling is therefore minimized.

## 2. Materials and Methods

### 2.1. Materials

Spray-drying experiments were conducted using the drug naproxen (Tokyo Chemical Industry CO., LTD., Tokyo, Japan) dissolved in acetone (Merck KGaA, Darmstadt, Germany). According to BCS classification, naproxen is classified as a Class IIa active substance and is thus dissolution-rate-limited in terms of its bioavailability [9]. Naproxen served as the model drug and was chosen mainly for its physical properties. The melting temperature is 155 °C [10] and the solubility of naproxen in acetone is high enough that a concentration in the spray liquid up to 20 wt-% in acetone did not lead to crystals in the feed tank. The excipient xylitol (Xylisorb 300, Roquette Pharma, Lestrem, France) was selected as the carrier matrix because of its function as a sweetening agent in both pharmaceutical and food processing. Xylitol has a melting temperature of 94 °C [11], allowing it to be melted without dissolving the separated naproxen particles. Furthermore, xylitol has a high water solubility (64 wt-% [12]), which should facilitate the dissolution of the solid dispersion.

### 2.2. Methods

#### 2.2.1. Preparation of Solid Dispersions in an MESP

**Figure 1:**
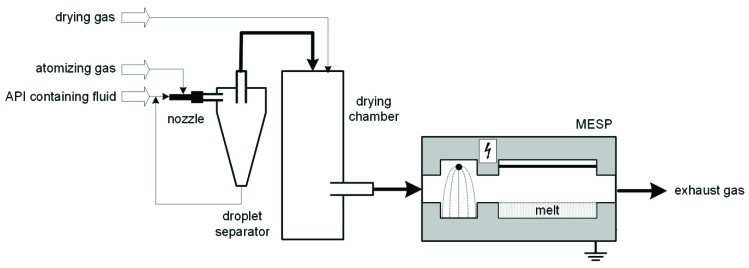
Schematic of the setup for spray drying experiments.

The spray drying apparatus, especially designed for the production of active ingredient particles in the submicron range, has already been described in more detail in previous work [13]. The schematic of the spray drying setup is shown in Figure 1.

To avoid explosive air mixtures, carbon dioxide was used for both spraying and drying. The prepared solution was sprayed via a two-fluid nozzle, which was operated with an HPLC pump (BlueShadow Pump 80P, KNAUER, Berlin, Germany) and a volume flow rate of 100 mL/min. The inert, atomization gas was used at a pressure of 3.5 bar and a mass flow of 3.7 kg/h. The aerosol produced was forced into a cyclone where large droplets (> 3µm) were separated out, while the small droplets entrained in the gas stream formed the conditioned aerosol that entered the drying section through the dip pipe. Carbon dioxide was supplied as a drying gas via a drying-gas distributor at an overpressure of 0.3 bar and a mass flow of 7.5 kg/h. Afterwards, the dried particles were charged and then separated into the molten xylitol in the electrostatic precipitator. After the melt cooled down, the solid dispersion was further processed.

#### 2.2.2. Characterization Methods

The obtained matrix/product was characterized with respect to particle size and dissolution behaviour. Initial investigations utilized scanning electron microscopy. Since drug particles could not be detected on the xylitol surface, the solidified melt was fractured, and images of the submicron particles were taken at the fracture surfaces with a scanning electron microscope (SEM) (Hitachi H-S4500 FEG, Krefeld, Germany) at 1 kV with a magnification of up to 40,000.

Additionally, the particle size distribution was measured with the Laser Diffraction Particle Sizer (Mastersizer 3000, Malvern Panalytical, Kassel, Germany) for wet dispersions by dissolving the xylitol matrix of the solid dispersion. A saturated naproxen solution was used to dissolve the matrix to prevent API dissolution during the measurement. No surfactant or disperser besides the Mastersizer pump and stirrer at 2000 rpm was necessary to measure the particle size distribution of the naproxen particles in the solid dispersion. The particle size measurement of the physical mixture as a reference was stabilized with sodium lauryl sulfate and dispersed with a gear disperser (IKA, Staufen, Germany) for 5 min at 9500 rpm.

Dissolution experiments were also performed with the obtained product and the physical mixture. They were tested using the United States Pharmacopeia (USP) Dissolution Apparatus 2 (DT 6, Erweka, Heusenstamm, Germany). The measurement conditions corresponded to the dissolution procedure of the USP [14]. The sample was dissolved in 900 mL of demineralized water at 37 °C using a stirrer speed of 50 rpm. A comparison to a physical naproxen-xylitol mixture was necessary to prove the effect on dissolution. The physical mixture was prepared according to the principal of geometric gradation in a laboratory mixer (Turbula T10B, W.A. Bachofen AG, Muttenz, Switzerland) at a maximum speed of 44 rpm. The UV/Vis spectrometer (Lambda 25, PerkinElmer, Waltham, USA) was used to quantify the active substance concentration in the solution. Calibration and measurements with naproxen were performed at a wavelength of 230 nm. The dissolution medium was pumped constantly through a flow cell and measured at a sampling rate of 10 s.

## 3. Results & Discussion

### 3.1. Electrostatic Precipitator Design

**Figure 2:**
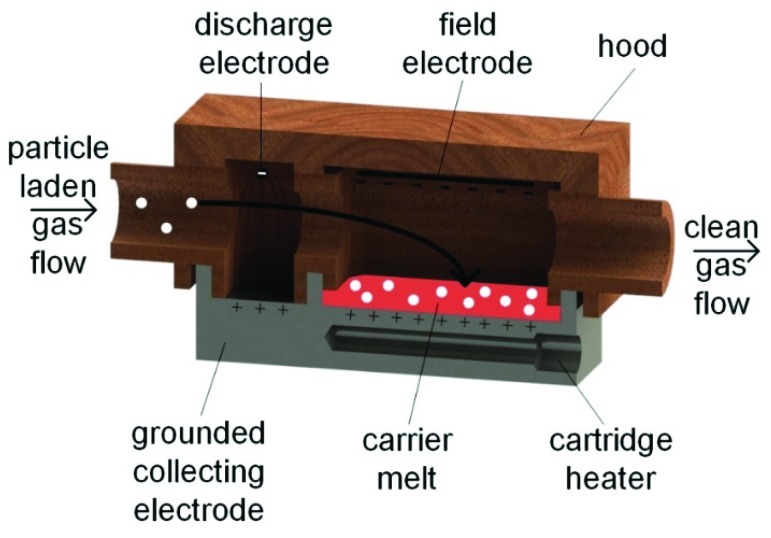
Schematic cross-section of the melt electrostatic precipitator.

Based on previous investigations of a dry two-stage electrostatic precipitator, a melt electrostatic precipitator prototype was developed. [15] The first stage of the MESP consists of a discharge electrode and a grounded counter electrode. Voltage is applied to the discharge electrode and increased until a corona discharge occurs. Particles located between the electrodes in the first stage are charged. In the second stage, no corona discharge occurs, so no further charging takes place. Instead, the already-charged particles are deflected in a second electric field towards the melt, which represents one of the two electrodes of different potential. The precipitation electrode or melt must be heated to keep the melt in a liquid state, otherwise the product would only precipitate on the surface of a solidified melt. In addition, the temperature must be controlled to prevent degradation of the carrier matrix or melting of the separated particles. For this purpose, the carrier substance xylitol was chosen because of its lower melting temperature than the deposited substance, but also for still being solid at room temperature. This substance was melted in the newly-developed device and subsequently loaded with submicron particles by electrostatic precipitation. Submicron particle production can only start once the carrier matrix has liquefied. A two-stage electrostatic precipitator improves the dry separation of submicron particles, because in the second stage, there is no turbulence due to electrostatic wind [15]. As is well known, the separation on a wet surface is significantly more efficient than in a dry ESP. However, the aim of this work is not to optimize the design, but to examine the feasibility of submicron particle separation into a melt. The MESP consists, as shown in Figure 2, of two areas in a stainless-steel metal block, which forms the grounded collecting electrode.

The prototype contains a cartridge heater (160W, Otom GmbH, Bräunlingen, Germany) and a temperature sensor (EF7, Otom GmbH, Bräunlingen, Germany). A controller (ETC 7420, ENDA, Istanbul, Turkey) ensures that the temperature of the melt is kept constant. The hood of the MESP is made of a hard, electrically insulating material. The insulating hood is equipped with a hole through which the loaded gas can flow. There are two holes for the wire of the discharge electrode for the first stage and for the field electrode in the second stage. Both the discharge and the field electrode are connected to a high voltage source (HPS 350W, iseg Spezialelektronik GmbH, Radeberg, Germany).

### 3.2. Experimental Results

**Figure 3:**
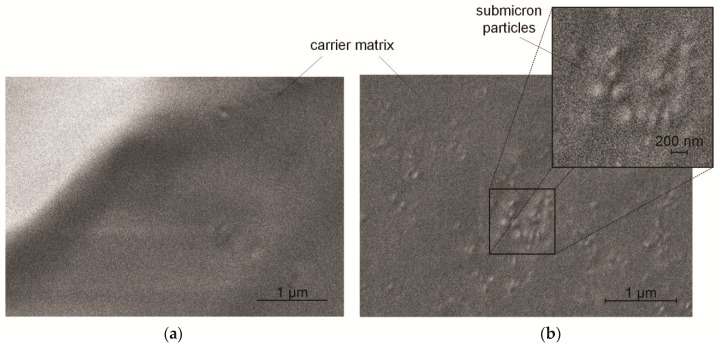
(**a**) Scanning electron microscope (SEM) image of the unloaded carrier surface; (**b**) and submicron naproxen particles in a xylitol carrier.

The prototype was evaluated by means of a spray-drying test for a period of 2 h. The solidified melt produced was examined in a scanning electron microscope. No particles were visible on the smooth surface of either the pure or the loaded xylitol carrier (Figure 3a). Figure 3b shows a picture of a fracture surface of the xylitol matrix carrying submicron naproxen particles.

In this image, isolated particles with a diameter of approximately 100–200 nm can be identified. This result shows that the formation of agglomerates can be avoided by deposition in a carrier melt. This observation is supported by the investigations of Kudryashova [7], where electrostatic charge reduces the contact angle in melts and enhances the wettability of submicron particles. Especially in comparison with the distribution of submicron particles in water, where poor wettability leads to a powder film on the water surface, the MESP is a promising device to produce solid dispersions containing isolated submicron particles.

**Figure 4:**
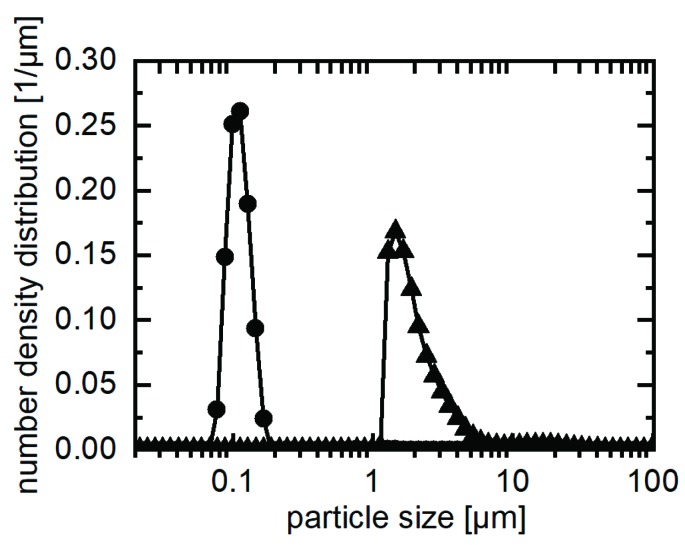
Particle size distributions of naproxen in xylitol processed in the melt electrostatic precipitator (●) and as a physical mixture of xylitol and naproxen for reference (▲).

The particle size distribution of spray-dried submicron particles was investigated in the preliminary stages by laser diffraction. Agglomerates can lead to very poor wetting and floating on the fluid surface. Since the particles were finely distributed in the xylitol, the wetting behaviour did not prevent dispersion of the particles in water. The measurement was therefore performed by dissolving the loaded xylitol crystal in a saturated solution of naproxen in water. This left the naproxen particles from the solid dispersion suspended and un-agglomerated in the measuring fluid. Figure 4 shows the size distribution of spray dried naproxen particles. The xylitol dissolved instantaneously and did not interfere with the measurement. For comparison, micronized naproxen as a physical xylitol mixture was measured and is also shown in Figure 4. The mean particle size in loaded xylitol is one order of magnitude smaller compared to a micronized interactive mixture.

**Figure 5:**
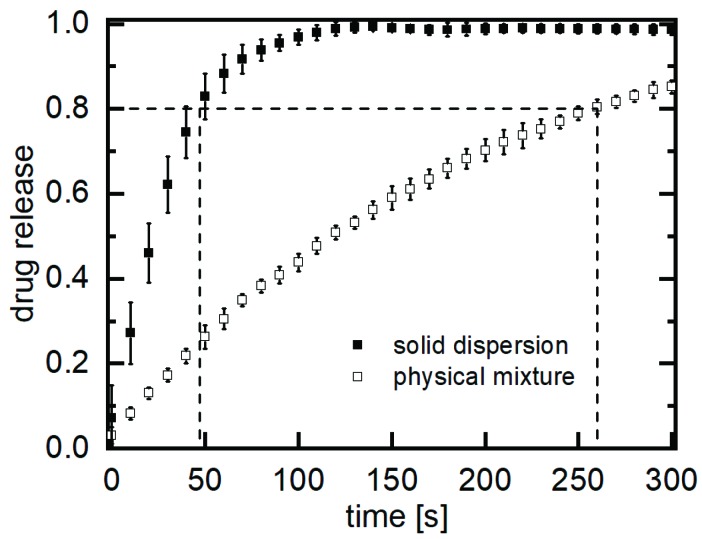
Dissolution profiles of submicron naproxen particles in xylitol [*t*_80_ = 46 s] and the unprocessed naproxen-xylitol physical mixture [*t*_80_ = 257 s], av ± CI, α = 0.05, *n* = 6.

In addition to the particle size analysis of the generated substance, the dissolution behaviour was also investigated. An improvement in water solubility can potentially lead to an increase in bioavailability. For this purpose, 1 g of the particle-laden xylitol with a load of about 0.01 wt.% was weighed and dissolved. As a reference, the same amount of the commercially available active ingredient naproxen was dissolved as a physical mixture under identical measuring conditions in order to investigate the effect on the dissolution. Figure 5 shows the dissolution kinetics of the active pharmaceutical ingredient, naproxen, embedded in xylitol compared to the physical mixture.

The improvement in the dissolution rate is evident from the slopes of the dissolution profiles. In less than 50 s, 80% of the entire dose of naproxen in the solid dispersion was released. In comparison, the release of 80% of the entire dose of naproxen in the physical mixture required 260 s, in this test.

These results prove the concept of melt electrostatic precipitation. It is possible to incorporate isolated particles into a melt by electrofiltration, significantly improving the dissolution rate in water.

## 4. Conclusions

A new type of electrostatic precipitator was developed for the production of solid dispersions containing submicron particles. A melt electrostatic precipitator prototype based on wet electrostatic precipitators was built and the feasibility was tested. A poorly water-soluble model API was spray dried and incorporated as submicron particles into a xylitol carrier matrix. It was possible to characterize the particle size distribution in the carrier as well as the improvement of the dissolution behavior. The drug release from the solid dispersion was five times faster than the release from a physical mixture of the micronized API and xylitol.

This prototype is promising with respect to the processability of cohesive powders, especially in the field of spray drying. The formation of agglomerates at the collecting electrode of the electrostatic precipitator could be avoided by directly depositing the particles into a carrier matrix.

## 5. Patents

Based on the work described in this manuscript, there exists a European patent application for a new electrostatic precipitator.
